# Effects of Local Tree Diversity on Herbivore Communities Diminish with Increasing Forest Fragmentation on the Landscape Scale

**DOI:** 10.1371/journal.pone.0095551

**Published:** 2014-04-17

**Authors:** Franziska Peter, Dana G. Berens, Nina Farwig

**Affiliations:** Department of Ecology - Conservation Ecology, Faculty of Biology, Philipps-University of Marburg, Marburg, Germany; Lakehead University, Canada

## Abstract

Forest fragmentation and plant diversity have been shown to play a crucial role for herbivorous insects (herbivores, hereafter). In turn, herbivory-induced leaf area loss is known to have direct implications for plant growth and reproduction as well as long-term consequences for ecosystem functioning and forest regeneration. So far, previous studies determined diverging responses of herbivores to forest fragmentation and plant diversity. Those inconsistent results may be owed to complex interactive effects of both co-occurring environmental factors albeit they act on different spatial scales. In this study, we investigated whether forest fragmentation on the landscape scale and tree diversity on the local habitat scale show interactive effects on the herbivore community and leaf area loss in subtropical forests in South Africa. We applied standardized beating samples and a community-based approach to estimate changes in herbivore community composition, herbivore abundance, and the effective number of herbivore species on the tree species-level. We further monitored leaf area loss to link changes in the herbivore community to the associated process of herbivory. Forest fragmentation and tree diversity interactively affected the herbivore community composition, mainly by a species turnover within the family of Curculionidae. Furthermore, herbivore abundance increased and the number of herbivore species decreased with increasing tree diversity in slightly fragmented forests whereas the effects diminished with increasing forest fragmentation. Surprisingly, leaf area loss was neither affected by forest fragmentation or tree diversity, nor by changes in the herbivore community. Our study highlights the need to consider interactive effects of environmental changes across spatial scales in order to draw reliable conclusions for community and interaction patterns. Moreover, forest fragmentation seems to alter the effect of tree diversity on the herbivore community, and thus, has the potential to jeopardize ecosystem functioning and forest regeneration.

## Introduction

The interaction between herbivorous insects and their host-plants play a key role for forest ecosystems. By feeding on plants, herbivores determine growth, reproduction, and survival of plants [1]. Thus, herbivory-induced leaf area loss (LAL) is considered an important factor for primary production, vegetation structure, the persistence of ecosystem functioning, and regeneration of plant-dominated ecosystems like forests [2]. However, the ongoing conversion of forest area to agriculturally used land poses a major threat to indigenous forests, forest-associated species, their interactions, and thus, the functioning of forest ecosystems [3].

Particularly, the increase of agriculturally used land at the expense of forest area results in small forest fragments that are spatially isolated by inhospitable landscape matrix [4]. Thus, anthropogenically driven forest fragmentation leads to habitat loss and decreasing habitat connectivity with consequences for the availability and the spatial distribution of resources on the landscape scale [5]. As a result, forest fragmentation entails direct implications for the composition of local communities and species distribution on a landscape scale, thereby altering interactions and trophic network patterns [5]. Finally, forest fragmentation has been suggested to ultimately imperil ecosystem functioning and forest regeneration [6]. Yet, recent research has revealed positive, negative, and neutral responses of herbivores to forest fragmentation [2], [7]–[9] as well as diverging effects on LAL [10], [11].

In addition to forest fragmentation on the landscape scale, plant diversity on the local habitat scale has been shown to be equally important for herbivore communities [12]. Plant diversity determines the number of different host-plant species as well as their proportionate availability. However, similarly to effects of forest fragmentation, studies showed diverging effects of tree diversity in forest habitats on herbivores as well as LAL [13]–[16], [17].

The inconsistencies in the effects of forest fragmentation and tree diversity on herbivores and LAL may be caused by interactive effects. Recent studies showed that environmental changes may not only act additively but also synergistically or antagonistically, leading to either an amplification or attenuation of the individual effects [18], [19]. As a result, the emerging effect cannot be interpreted by separately focussing on single factors or by adding together the individual effects [19]. Thus, de Sassi et al. [18] emphasize the need to consider both main effects and interactive effects of multiple, relevant, co-occurring factors in concert. Assuming interactive effects of forest fragmentation on the landscape scale and tree diversity on the local habitat scale may well explain the diverging responses of herbivores and LAL throughout the body of studies. For instance, a recent study of Roesch et al. [7] showed an interactive effect of habitat isolation and plant species richness on a generalist leafhopper community. In this study, species richness of generalist leafhoppers increased with increasing plant species richness while the magnitude of the positive effect was higher in connected compared to isolated grassland habitats. Hence, forest fragmentation on the landscape scale and tree diversity on the local habitat scale may interact in a synergistic or antagonistic manner. Consequently, the direction and magnitude of the emerging effect of forest fragmentation and tree diversity on the herbivore community, and on LAL may vary considerably. Yet, interactive effects of environmental changes that act on different spatial scales are still poorly understood. Moreover, it is unknown whether interactive effects of forest fragmentation and tree diversity show a consistent pattern for the entire herbivore community.

Therefore, the aim of our study was to investigate the interactive effects of forest fragmentation on the landscape scale and tree diversity on the local habitat scale on herbivore communities and on LAL. Since previous studies reported inconsistent results regarding effects of the two environmental factors we did not corroborate hypotheses regarding the character of the main effects of forest fragmentation and tree diversity. Yet, similarly to the study of Roesch et al. [7] we hypothesized a change in the effect of tree diversity on the herbivore community along the gradient of forest fragmentation. Furthermore, depending on the direction and magnitude of the emerging interactive effect of forest fragmentation and tree diversity on the herbivore community, we expected LAL to change correspondingly.

## Methods

### Study Region

The study was conducted within and around the Oribi Gorge Nature Reserve (OGNR; 30°40′ to 30°45′ S and 30°10′ to 30°18′ E; 1881 ha) in southern KwaZulu-Natal, eastern South Africa. The necessary research permits for the OGNR were obtained from Ezemvelo KZN Wildlife. All study sites outside OGNR were on private property of local farmers, who granted us access to their land. The average rainfall of the region ranges from 570 to 1625 mm per year with a maximum in summer (October to March), and the average daily temperature ranges from 13 to 23°C [20]. The study region is characterized by a large proportion of agriculturally used land mainly comprising sugar cane. This agricultural landscape matrix is interspersed with indigenous forest, predominantly forest remnants and only a small number of continuous forests. The regional indigenous forest type is scarp forest constituting a mixture of Afrotemperate and Indian Ocean coastal belt forest [21].

### Forest Fragmentation and Tree Diversity

Forest fragmentation on the landscape scale entails several consequences such as the loss of forest area, decreasing fragment size, and increasing isolation of forest remnants [22]. Studies assessing effects of spatial changes on the landscape scale determined the area of the respective land-use or habitat type within a given landscape to be the most important determinant for the composition and structure of biotic communities [22], [23]. Therefore, we defined forest fragmentation as the ratio of agriculturally used area to the total area within a given landscape. We selected ten study sites within continuous and fragmented indigenous scarp forests that showed an increasing degree of forest fragmentation within 1000 m radii around the centres of the study sites. We are aware that the response of herbivores to landscape changes is scale-dependent [23]. However, we chose the 1000 m radius as landscape effects on herbivores and herbivory have been shown to be strongest on a spatial scale between 500 and 1500 m [24]. Furthermore, forest fragmentation for the 1000 m radius was highly correlated with forest fragmentation for other radii (500–1500 m; Pearson correlation: r >0.96; n = 10; P-value <0.001 in all cases), and the choice of the 1000 m radius should therefore not substantially influence our findings. Across the ten study sites forest fragmentation ranged from 0.08 to 0.87. Mean pair-wise distances between study sites ranged from 1,400 to 20,700 m (9,500±5,400 m; mean ± standard deviation (SD) throughout). Calculations of forest fragmentation were based on KwaZulu-Natal Land Cover data from Ezemvelo KZN Wildlife ([25]; resolution: 20 m×20 m) using ArcGIS (9.3.).

On the local habitat scale we defined tree diversity as the index of Shannon diversity. To asses tree diversity we randomly chose five plots (10 m×10 m each) within each forest study site adding up to a total area of 500 m^2^ per study site. The distances among plots and between the plots and forest edges were at least 10 m. Within the plots we identified all trees [26] higher than 2 m and calculated tree diversity per study site. In total, we recorded 2519 tree individuals from 147 tree species and 53 plant families. Species from the family Rubiaceae were most common (16.4%, 20 species), followed by species of Euphorbiaceae (12.8%, 9 species) and Sapotaceae (9.5%, 2 species). Tree diversity ranged from 1.72 to 3.22 comprising 17 to 48 different tree species. Forest fragmentation and tree diversity showed moderate but non-significant correlation (Pearson correlation: r = −0.50; n = 10; P-value = 0.138). We ultimately evaluated the potential collinearity and related goodness of our statistical results by calculating the Variance Inflation Factor (VIF) for the regression models ([27]; see statistical analyses for details).

### Choice of Tree Species and Sampling of Herbivores

To assess plant-herbivore interactions for a representative set of the tree community and the associated herbivore communities we selected the most abundant tree species per study site (focal tree species, hereafter). Thus, the selection was based on the availability of tree species at individual study sites. We included every tree species of which we found 15 individuals per study site within a range of about 50 m×50 m. Across the ten study sites we selected 67 focal trees with 29 different tree species from 21 families (see Table S1). The number of focal tree species ranged from five to nine tree species per study site and accounted for 47 to 78% of the tree community per study site (63±10%). Due to differences in the abundance distribution of tree species within the study sites the composition of the set of focal tree species varied across the study sites. In order to account for the variation in tree species identity, we included a phylogenetic eigenvector into our statistical analysis.

For the collection of herbivores we applied standardized beating samples from the end of March to the middle of April 2012. To ensure the collection of sufficient numbers of herbivores we collected beating samples from 15 individuals per focal tree species per study site and pooled these samples for further analyses. The height of the selected tree individuals ranged from 2 to 3 m. The standardized beating technique involved ten beatings with a wooden club against one randomly selected part of the tree. We collected the beating samples in a plastic funnel connected to a water-filled container. We separated the insects from unintended by-catch and debris and stored them in small flasks (containing 70% ethanol). We identified the insects to the lowest taxonomic level possible (mainly family and genus level; [28]) and further discriminated them into morphospecies. Literature and expert knowledge for species taxonomy was relatively coarse. However, as the taxonomic resolution is equal across the insect orders of our beating samples and study sites, the coarse taxonomic resolution should not affect our results. Finally, we determined the morphospecies that are herbivorous (including omnivorous families within Coleoptera). The relative abundance of herbivorous insects ranged from 0 to 80% per focal tree species per study site (27.6±17.6%). For further analyses we only considered herbivorous insects.

### Herbivore Community Composition, Herbivore Abundance, and Number of Herbivore Species

To analyse changes in the herbivore community composition due to forest fragmentation and tree diversity we compiled a matrix with abundances of herbivores per focal tree species per study site and applied a Hellinger-transformation. Based on the transformed abundance matrix we established a dissimilarity matrix by calculating Bray-Curtis distances. In addition to forest fragmentation and tree diversity, we included a spatial component to account for spatial autocorrelation of the occurrence of herbivore species [24]. We derived the spatial component by applying a Principal Coordinates of Neighbourhood Matrix analysis (PCNM) on the abundance matrix. From a matrix of spatial eigenvectors we selected the most significant eigenvector by using stepwise forward selection with alpha = 0.01 and 9,999 permutations (PCNM1: adj. R^2^ = 0.04; P-value = 0.001).

The abundance and the diversity of herbivores have been suggested to affect the feeding pressure per plant individual [29]. Thus, using the abundance matrix of herbivore species per tree species per study site we calculated herbivore abundance and the effective number of herbivore species (exponent of Shannon diversity; number of herbivore species, hereafter). We calculated the two response variables on tree species-level (i.e. for each focal tree species per study site) to account for diverging responses of herbivores to the identity of focal tree species.

### Estimation of Leaf Area Loss

Leaf area loss (LAL) was defined as the percentage of lost photosynthetically active leaf area due to leaf-chewing. As our herbivore samples contained no leaf-mining larvae we assumed the completion of the larval stage of most leaf-mining insects, and thus, excluded LAL due to leaf-mining. We visually estimated LAL in the field for 30 randomly chosen leaves of ten tree individuals per focal tree species within each study site and calculated the mean percentage per tree species per study site.

### Statistical Analysis

To analyse effects of the spatial component, forest fragmentation, and tree diversity on the herbivore community composition we performed non-parametric permutational Multivariate Analysis of Variance (perMANOVA [30]) using the transformed abundance matrix. The perMANOVA partitions dissimilarities across the chosen terms of predictor variables, here the spatial component, forest fragmentation, and tree diversity. This analysis uses permutations on raw data within a specified group to evaluate significances of the predictors. In a perMANOVA the respective predictor variables are evaluated sequentially as determined by the formula interface, and thus, significances may change depending on the order of terms in the model formula. Therefore, we fitted four separate models, shuffled the last predictor term in the model formula, and took the statistics from the predictor variable of the last term.

To explore the causal relationships between forest fragmentation, tree diversity, herbivore abundance, number of herbivore species, and LAL we conducted a path analysis. Accounting for the nested structure of our data we applied path analysis after Shipley’s d-separation method [31] and used linear mixed-effects models (LMER). As random effects we assigned either, both, study site and focal tree species identity, or only the former as random effects depending on their individual values of explained variance for the respective models. To enable the comparison of the effect sizes of the fixed effects we applied z-transformation. The estimate of tree diversity for one study site constituted an outlier. Yet, removing the outlier did not change the results, and thus, we retained data points belonging to this study site. To ensure normal distribution of response variables we applied ln-transformation throughout. We fitted the LMERs using restricted maximum likelihood (REML) and derived the P-values from Markov Chain Monte Carlo sampling (pMCMC).

**Figure 1:**
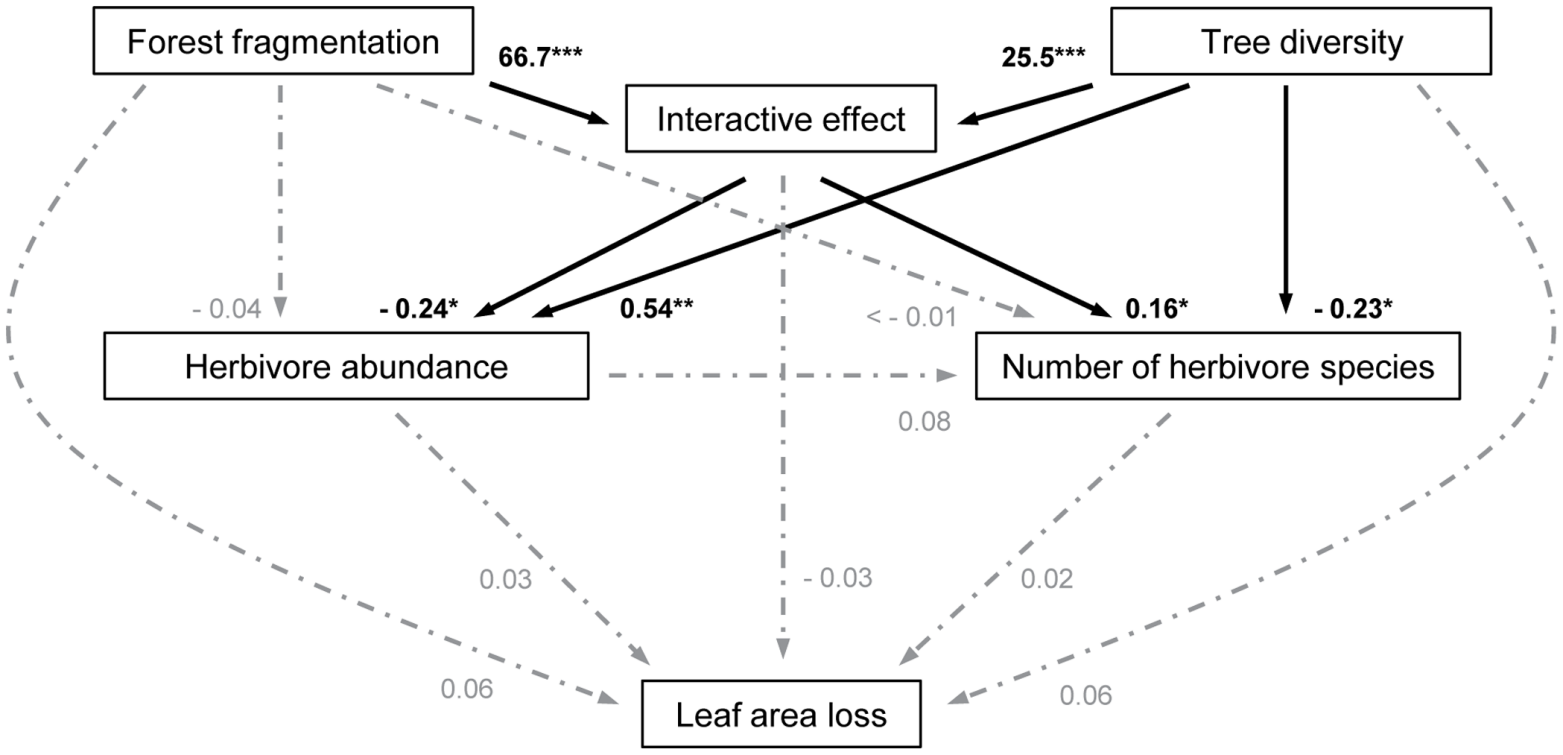
Path model for relationships between forest fragmentation, tree diversity, herbivore community, and leaf area loss. Causal relationships between forest fragmentation, tree diversity, herbivore abundance, number of herbivore species, and leaf area loss. Values next to arrows give effect estimates; black estimates and solid arrows show significant effects, and stars demark the significance level (pMCMC: 0.050< * >0.010< ** >0.001< *** >0.000).

Following Shipley’s path analysis we compiled a set of initial models based on the causal relationships between forest fragmentation, tree diversity, herbivore abundance, number of herbivore species, and LAL (Fig. 1). The first model included the effect of forest fragmentation and tree diversity on their combined interactive term. The following two models included the main effects and interactive effects of forest fragmentation and tree diversity on herbivore abundance and the number of herbivore species. To account for the potential causal relationship between herbivore abundance and the effective number of herbivore species we additionally included herbivore abundance as predictor for the number of herbivore species in the latter model. The fourth model included the main effects and interactive effect of forest fragmentation and tree diversity, as well as the effects of herbivore abundance and the number of herbivore species on LAL.

To evaluate the potential collinearity of the two fixed effects forest fragmentation and tree diversity and the related goodness of our results we calculated the Variance Inflation Factor (VIF) for the models investigating the effects on herbivore abundance, number of herbivore species, and LAL. The VIF represents the overall correlation of each predictor with all others in the same model [27]. Generally, a VIF above 10 indicates “severe” collinearity while values below 4 have been suggested to be uncritical. The VIF values for all models were below the critical threshold, (herbivore abundance: <1.6; number of herbivore species: <1.5; LAL: <1.8). Thus, we are confident that the potential collinearity of forest fragmentation and tree diversity did not affect the results of our study.

Based on the significances we derived from the initial four models, we subsequently applied d-separation to test each hypothesized conditional independency separately using the LMERs. We thus obtained the probability that the partial slope of the dependent variable was significantly different from zero. Finally, we combined and tested the probabilities of all independence claims using C-Statistics [31]. The result of the Chi^2^-test supported the causal model assumptions (C = 7.27; df = 16; P-value = 0.968).

In order to account for the different sets of focal tree species across our study sites, we included a phylogenetic eigenvector in our analyses. We derived the phylogenetic eigenvector by firstly generating a phylogenetic tree including all the tree species we sampled during the vegetation monitoring. We generated the phylogenetic tree using Phylomatic version 3 (http://phylodiversity.net/phylomatic/) based on a megatree (R20120829) provided by the online program. Using the application Phylocom version 4.2 and the internal megatree of the program with given branch lengths (based on the divergence in DNA sequence data [32]) we adjusted the branch lengths of our phylogenetic tree. Based on this adjusted phylogenetic tree we calculated pairwise phylogenetic distances between all the tree species and compiled a distance matrix including the focal tree species per study site (in rows) and the phylogenetic distances to the rest of the focal tree species (in columns). Next, we applied a Principal Coordinates of Neighbourhood Matrix analysis (PCNM) on this distance matrix to generate a matrix of eigenvectors. With stepwise forward selection with alpha = 0.05 and 9,999 permutations, we selected one phylogenetic eigenvector for the individual response variables herbivore community composition, herbivore abundance, number of herbivore species, and LAL. However, the individual phylogenetic eigenvectors had no effect on the tested response variables throughout (pMCMC >0.095). Thus, we concluded that the identity of the chosen focal trees did not affect our results.

All statistical analyses were done using Software R version 2.14.2 [33] including packages ‘vegan’ [34] for calculation of the number of herbivore species and the perMANOVA, ‘packfor’ [35] for forward selection, ‘lme4’ [36] for calculating LMERs, and ‘languageR’ [37] for extracting pMCMC-values and plotting the interactive effects of LMERs.

## Results

### Herbivore Community Composition, Herbivore Abundance, and Number of Herbivore Species

Across the study sites we sampled 763 herbivorous insects (87 morphospecies) from seven orders with Coleoptera being most abundant (83.6%; Curculionidae 70.8%), followed by Orthoptera (10.6%), Blattodea (2.5%), Hemiptera (2.0%), Hymenoptera (0.8%), Diptera and Phasmatodea (0.3% each).

**Figure 2:**
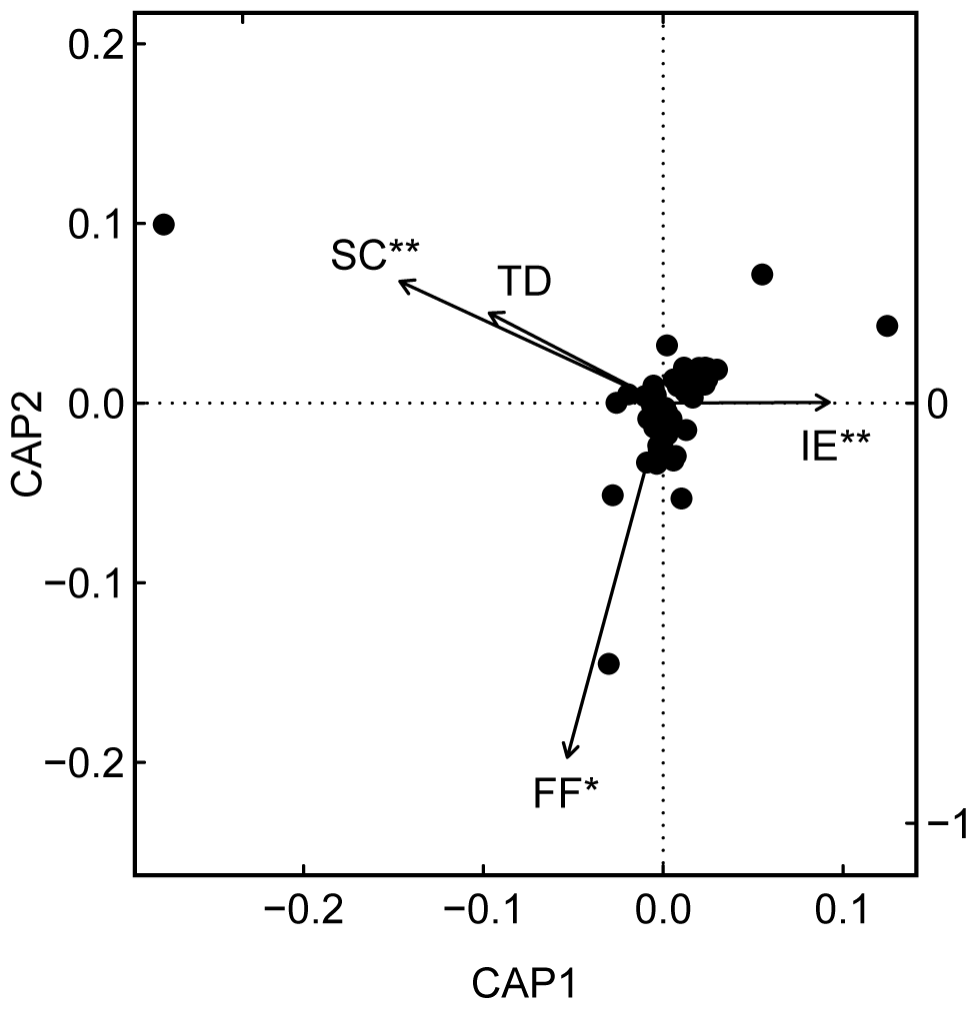
Effects of forest fragmentation and tree diversity on the community composition of herbivores. Ordination plot of herbivore species per tree species per study site along the spatial component (SC), the gradients of forest fragmentation (FF) and tree diversity (TD), and their interactive effect (IE). Black points display species scores (n = 87) and stars demark the significance level (pMCMC: 0.050< * >0.010< ** >0.001< *** >0.000). We used a Constrained Analysis of Principal Coordinates (CAP) for visualization only as Software R does not provide a function to plot results of the perMANOVA.

Herbivore community composition per tree species per study site was related to the spatial component (R^2^ = 0.04; F_1,62_ = 3.42; P-value = 0.006; Fig. 2) and changed along the gradient of forest fragmentation (R^2^ = 0.06; F_1,62_ = 4.44; P-value = 0.043), but was not affected by tree diversity (R^2^ = 0.04; F_1,62_ = 3.02; P-value = 0.895). However, forest fragmentation and tree diversity interactively affected herbivore community composition per tree species per study site (R^2^ = 0.05; F_1,62_ = 4.14; P-value = 0.005). The observed changes were mainly driven by four species of the family Curculionidae (Coleoptera) that dominated the herbivore community throughout (58.5±31.4% per tree species per study site). Interestingly, though abundances of Curculionidae were comparably high across all study sites, different species dominated the respective herbivore communities per tree species per study site.

**Figure 3:**
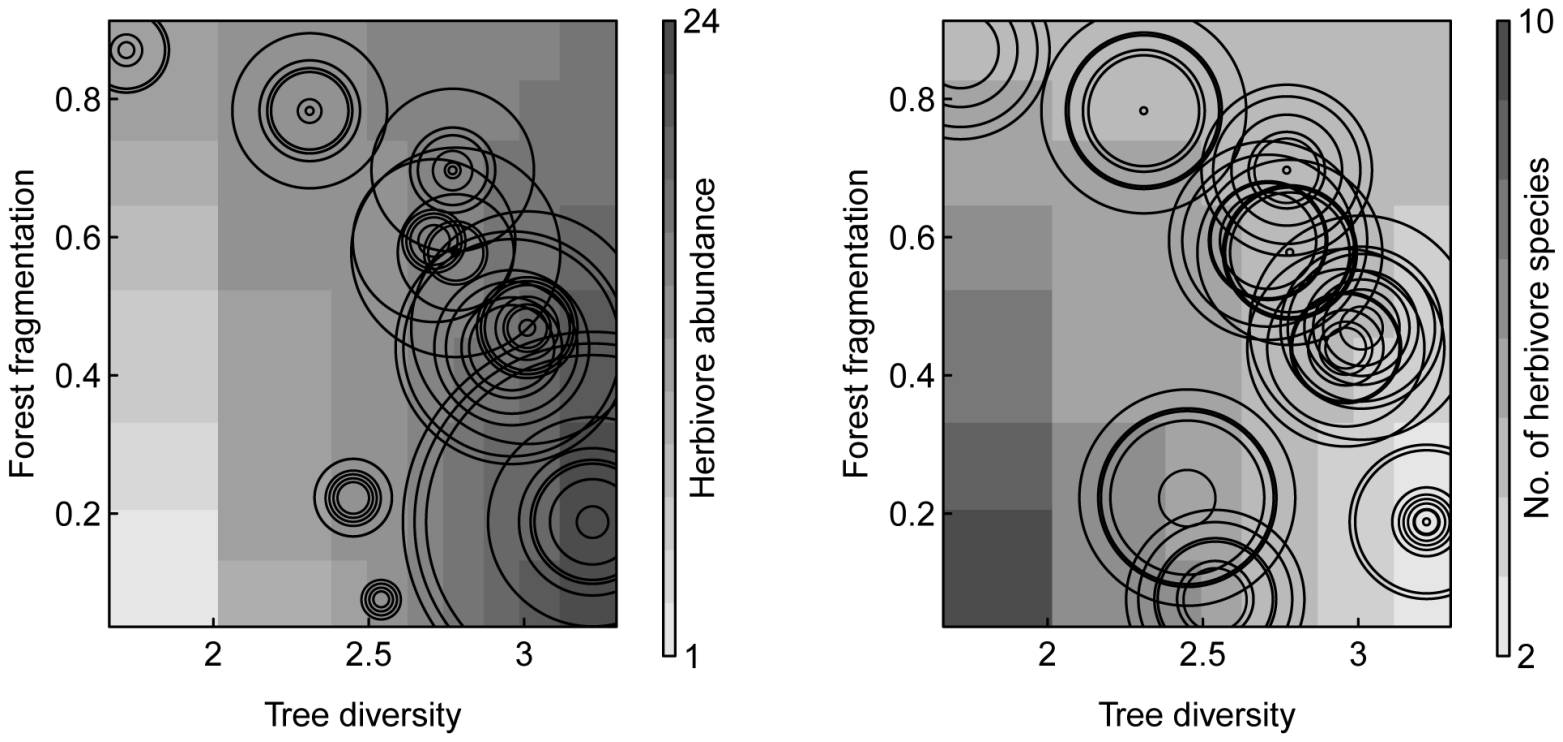
Effects of forest fragmentation and tree diversity on herbivore abundance and number of herbivore species. Light to dark grey shaded areas depict low to high values for (left panel) herbivore abundance and (right panel) number of herbivore species per tree species per study site based on model fit; small to large radii of circles depict low to high values of original data for herbivore abundance and number of herbivore species per tree species per study site.

Herbivore abundance per tree species ranged from 1 to 49 (11.4±10.6; n = 67). Forest fragmentation did not affect herbivore abundance (Fig. 1). However, herbivore abundance increased with increasing tree diversity. Furthermore, forest fragmentation and tree diversity had an interactive effect on herbivore abundance (Fig. 3). Herbivore abundance only increased with increasing tree diversity in slightly fragmented forests whereas the effect diminished with increasing forest fragmentation. As herbivore abundance was not correlated with the number of tree individuals per study site we were able to exclude changes in herbivore abundance as a result of changes in the number of tree individuals (Pearson correlation: r = 0.04; n = 67; P-value = 0.741).

The number of herbivore species per tree species ranged from 1.0 to 7.5 (3.2±1.7; n = 67). Forest fragmentation did not affect the number of herbivore species per tree species (Fig. 1). In contrast, the number of herbivore species per tree species significantly decreased with increasing tree diversity. Yet, similarly to the interactive effect on herbivore abundance, the effect of tree diversity on the number of herbivore species was only present in slightly fragmented forests (Fig. 3). Furthermore, herbivore abundance and the number of herbivore species were not related to each other (Fig. 1).

### Leaf Area Loss

LAL per tree species due to leaf chewing ranged from 0.7 to 26.0% (7.9±0.7%; n = 67). Forest fragmentation and tree diversity had no main or interactive effects on LAL (Fig. 1). Furthermore, neither herbivore abundance nor the number of herbivore species per tree species per study site affected LAL per tree species.

## Discussion

The results of our study showed that the community composition of herbivores changed due to interactive effects of forest fragmentation and tree diversity. Moreover, our results indicated an increase in herbivore abundance and a decrease in the number of herbivore species with increasing tree diversity for slightly fragmented forests. In contrast, in highly fragmented forests neither herbivore abundances nor the number of herbivore species changed along the gradient of tree diversity. Yet, despite the effects of forest fragmentation and tree diversity on herbivore abundance and the number of herbivore species we could not detect a link to LAL.

### Main and Interactive Effects of Forest Fragmentation and Tree Diversity

The emerging pattern of the effects of forest fragmentation on the landscape scale and of tree diversity on the local habitat scale revealed two key aspects why studies need to consider interactive effects of environmental changes. Firstly, both forest fragmentation and tree diversity did not always show main effects on the herbivore community despite significant interactive effects. Thus, the effect of forest fragmentation and tree diversity on the herbivore community only became apparent through the interactive effects of both environmental factors. Furthermore, even after removing the interactive term from the model regressions the main effects did not become significant. Hence, if studies do not incorporate potential interactive effects of environmental changes they may be prone to overlook individual effects and draw wrong conclusions regarding their ecological significance [18]. Secondly, while herbivore abundance and the number of herbivore species were affected by increasing tree diversity in slightly fragmented forests, both response variables did not change along the gradient of tree diversity in highly fragmented forests. Hence, the direction and the magnitude of the effect of one environmental factor may strongly depend on the specification of other environmental factors. Thus, according to our expectations our findings support that studies showing diverging responses of herbivores to changes in either forest fragmentation on the landscape scale or tree diversity on the local habitat scale may be biased by not accounting for potential interactive effects.

### Herbivore Community Composition, Herbivore Abundance, and Number of Herbivore Species

The spatial component, forest fragmentation, and tree diversity significantly affected the community composition of herbivores (Fig. 2). The Curculionidae, which accounted for the majority of herbivores, were highly abundant across all study sites. However, results indicated a species turnover within this family with forest fragmentation and tree diversity. The emerging pattern in the species turnover of herbivore communities along the gradient of forest fragmentation suggests a selection according to body size (measured as dry weight), and thus, dispersal ability. More specifically, Curculionidae showed specific shifts in body size with forest fragmentation: The mean dry weight of Curculionidae per tree species per study site ranged from 0.1 to 2.6 mg (0.8±0.6 mg) and increased with increasing forest fragmentation (pMCMC = 0.014; estimate = 0.04). Thus, species that dominated the herbivore community in slightly fragmented forests were smaller and were gradually substituted by larger species with increasing forest fragmentation (>20-fold increase in dry weight). This positive relationship between forest fragmentation and body size is congruent with findings of other studies (e.g. [38]) and may be explained by environmental filtering of the herbivore community based on species-specific dispersal abilities [39], [40]. Dispersal ability is positively linked to body size [38], and thus, particularly large species may show a higher capability to traverse inhospitable matrices between isolated forest fragments [5], [40]. In contrast, smaller species may be more susceptible to forest fragmentation and experience a decline in migration and recolonization events [22] resulting in comparably small population sizes on the local habitat scale [41]–[43].

At the same time, the environmental filter of forest fragmentation for higher dispersal ability may explain the interactive effects of forest fragmentation and tree diversity on overall herbivore abundance and the number of herbivore species. While herbivore abundance increased with increasing tree diversity in slightly fragmented forests, this effect diminished with increasing forest fragmentation. Thus, similarly to findings of Roesch et al. [7], the spatial isolation of herbivore communities in fragmented forests may have hampered an overall increase of herbivore abundances with increasing tree diversity due to lower migration and recolonization events. Analogous, the decrease in the number of herbivore species with increasing tree diversity was only apparent in slightly fragmented forests. Large and highly mobile herbivore species that show a low susceptibility to forest fragmentation on the landscape scale are unlikely to respond to differences in tree diversity on the local habitat scale [44]. Thus, environmental filtering of the herbivore community by forest fragmentation on the landscape scale may have driven the species turnover related to body size, and simultaneously, may have circumvented the effects of tree diversity on the local habitat scale on herbivore abundance and the number of herbivore species in highly fragmented forests.

In contrast to the underlying mechanism of the effect of forest fragmentation, the effect of tree diversity on the herbivore community may be explained by species-specific differences in host-tree preferences and diet breadth [12]. Increased tree diversity has been suggested to provide a higher number of supplementary or even more appropriate host-tree species within close proximity (e.g. [14]). In turn, particularly generalist species may benefit from dispersing across the increased variety of different tree species [14] in order to feed on their preferred host-tree species, to reduce niche overlap and competitive pressure, or to locate enemy-free space [12]. As a result, increased tree diversity may support higher abundances of particularly generalist species [45]. This assumption corresponds to our finding that Curculionidae species that were related to study sites with high tree diversity were highly abundant on all focal tree species. In contrast, Curculionidae species that were associated with study sites showing low to medium tree diversity were only present on a subset of focal tree species and were less abundant throughout. The dispersal of certain herbivore species across the tree community with increasing tree diversity and the related increase in their individual abundances may simultaneously explain the overall increase in herbivore abundance with increasing tree diversity. Moreover, this dispersal of certain herbivore species in highly diverse forests may have also caused the reduction in the number of herbivore species per focal tree species. Thus, our results suggest that increased tree diversity promotes higher abundances of particularly generalist herbivores and leads to lower numbers of herbivore species per tree species in slightly fragmented forests.

### Leaf Area Loss

Despite the interactive effects of forest fragmentation and tree diversity on the herbivore community we could not detect a link to LAL. This discrepancy may be explained by two not mutually exclusive factors. Firstly, LAL due to leaf-chewing represents an accumulation of feeding events throughout the whole season while our arthropod sampling represented only a “snapshot” of the current state of the arthropod community during the entire season of herbivore activity [46]. Yet, diverse subtropical forests encompass a huge variety of herbivore species with different patterns regarding their life cycle and related changes in their feeding habits [47] leading to population fluctuations and changes in host-tree choice during their ontogenetic development [48]–[50]. As a result, a turnover in the herbivore community composition throughout the whole season [46], [51] may have compromised the conclusion whether effects of forest fragmentation and tree diversity on the herbivore community translate into changes in LAL. Secondly, the communities of herbivores on the respective focal tree species may have contained a certain proportion of tourist species that did not necessarily feed on the individual tree species, and thus, may not have contributed to the respective degree in LAL. Hence, future studies should incorporate seasonal changes of herbivore communities and ensure the trophic interaction between herbivores and the focal tree species to further evaluate the interactive effects of environmental changes on the landscape and the local habitat scale on LAL.

## Conclusion

With the interactive effects of forest fragmentation on the landscape scale and tree diversity on the local habitat scale on the herbivore community we highlight the importance to consider joint effects of environmental changes across different spatial scales in general. Strikingly, tree diversity determined patterns of the herbivore communities while the magnitude of the effect on the herbivore community was altered by the degree in forest fragmentation. Based on our data, we could not confirm whether changes in the herbivore community due to forest fragmentation and tree diversity translate into changes in leaf area loss. Yet, findings of our study provide evidence that environmental changes across spatial scales may have the potential to ultimately affect primary production, vegetation structure, the persistence of ecosystem functioning, and the regeneration of forests via altered plant-herbivore interactions.

## Supporting Information

Table S1 **Focal tree species across the ten study sites.** We selected 67 focal trees across the ten study sites belonging to 29 different tree species from 21 families; selection was based on the proportionate availability of tree species at the individual study sites; we included every tree species of which we found 15 individuals per study site within a range of about 50 m×50 m; tree species are sorted by frequency of occurrence across the study sites in descending order; the two last rows give the number of selected focal tree species per study site and their overall proportion as part of the tree community per study site.
(DOCX)
