# Supplementary material for: Effects of Local Tree Diversity on Herbivore Communities Diminish with Increasing Forest Fragmentation on the Landscape Scale
Source: PLoS One. 2014 Apr 17;9(4):e95551. doi: 10.1371/journal.pone.0095551 (PMC3990639; doi:10.1371/journal.pone.0095551)
Supplement: Table S1 — Focal tree species across the ten study sites. We selected 67 focal trees across the ten study sites belonging to 29 different tree species from 21 families; selection was based on the proportionate availability of tree species at the individual study sites; we included every tree species of which we found 15 individuals per study site within a range of about 50 m×50 m; tree species are sorted by frequency of occurrence across the study sites in descending order; the two last rows give the number of selected focal tree species per study site and their overall proportion as part of the tree community per study site. (DOCX) [file pone.0095551.s001.docx]

**Supporting Information**

**Table 1: Focal tree species across the ten study sites.** We selected 67 focal trees across the ten study sites belonging to 29 different tree species from 21 families; selection was based on the proportionate availability of tree species at the individual study sites; we included every tree species of which we found 15 individuals per study site within a range of about 50 m x 50 m; tree species are sorted by frequency of occurrence across the study sites in descending order; the two last rows give the number of selected focal tree species per study site and their overall proportion as part of the tree community per study site.

| **Tree species** | **Tree family** | **Study site** | | | | | | | | | | **∑** |
| --- | --- | --- | --- | --- | --- | --- | --- | --- | --- | --- | --- | --- |
|  |  | **1** | **2** | **3** | **4** | **5** | **6** | **7** | **8** | **9** | **10** |  |
| *Monanthotaxis caffra* | Annonaceae |  | x | x | x | x | x | x | x | x | x | 9 |
| *Englerophytum natalense* | Sapotaceae |  | x |  | x | x | x | x | x | x | x | 8 |
| *Eugenia natalitia* | Myrtaceae |  |  | x |  | x |  | x | x | x |  | 5 |
| *Cassipourea malosana* | Rhizophoraceae |  |  | x |  |  |  |  | x | x | x | 4 |
| *Drypetes arguta* | Euphorbiaceae |  |  | x |  |  | x |  |  | x | x | 4 |
| *Peddiea africana* | Thymeleaceae |  | x |  |  | x |  | x |  | x |  | 4 |
| *Allophylus dregeanus* | Sapindaceae | x | x |  | x |  |  |  |  |  |  | 3 |
| *Chionanthus foveolatus tomentellus* | Oleaceae |  |  |  |  |  | x | x | x |  |  | 3 |
| *Rapanea melanophloeos* | Myrsinaceae | x | x |  |  | x |  |  |  |  |  | 3 |
| *Memecylon natalense* | Melastomataceae |  |  | x |  |  |  |  | x |  |  | 2 |
| *Ochna arborea* | Ochnaceae |  |  | x |  |  |  |  |  |  | x | 2 |
| *Uvaria caffra* | Annonaceae |  |  |  |  |  |  | x |  | x |  | 2 |
| *Xymalos monospora* | Monimiaceae |  |  |  | x | x |  |  |  |  |  | 2 |
| *Brachylaena uniflora* | Asteraceae |  |  |  |  |  |  | x |  |  |  | 1 |
| *Bridelia micrantha* | Euphorbiaceae | x |  |  |  |  |  |  |  |  |  | 1 |
| *Cassipourea gummiflua* | Rhizophoraceae |  |  |  | x |  |  |  |  |  |  | 1 |
| *Cryptocarya transvaalensis* | Lauraceae | x |  |  |  |  |  |  |  |  |  | 1 |
| *Cryptocarya woodii* | Lauraceae |  |  |  |  |  |  |  |  | x |  | 1 |
| *Euclea natalensis natalensis* | Ebenaceae |  |  |  |  |  |  |  |  | x |  | 1 |
| *Gymnosporia harveyana* | Celastracea |  |  |  |  |  |  |  | x |  |  | 1 |
| *Halleria lucida* | Scrophulariaceae | x |  |  |  |  |  |  |  |  |  | 1 |
| *Nectaropetalum capense* | Erythroxylaceae |  | x |  |  |  |  |  |  |  |  | 1 |
| *Rothmannia globosa* | Rubiaceae |  |  |  |  |  | x |  |  |  |  | 1 |
| *Strychnos henningsii* | Strychnaceae |  |  | x |  |  |  |  |  |  |  | 1 |
| *Strychnos usambarensis* | Strychnaceae |  |  |  |  |  |  |  |  |  | x | 1 |
| *Syzygium guineense guineense* | Myrtaceae | x |  |  |  |  |  |  |  |  |  | 1 |
| *Teclea natalensis* | Rutaceae |  |  |  |  |  |  |  | x |  |  | 1 |
| *Tricalysia capensis capensis* | Rubiaceae |  |  |  | x |  |  |  |  |  |  | 1 |
| *Tricalysia lanceolata* | Rubiaceae |  |  |  |  | x |  |  |  |  |  | 1 |
| **Number of focal tree species per study site** | | 6 | 6 | 7 | 6 | 7 | 5 | 7 | 8 | 9 | 6 | 67 |
| **Proportion of focal tree species within tree community [%]** | | 78 | 61 | 74 | 70 | 55 | 47 | 59 | 63 | 52 | 71 |  |
